# Population Analysis of *Escherichia coli* Sequence Type 361 and Reduced Cefiderocol Susceptibility, France

**DOI:** 10.3201/eid2909.230390

**Published:** 2023-09

**Authors:** Agnès B. Jousset, Laura Bouabdallah, Aurélien Birer, Isabelle Rosinski-Chupin, Jean-François Mariet, Saoussen Oueslati, Cécile Emeraud, Delphine Girlich, Philippe Glaser, Thierry Naas, Rémy A. Bonnin, Laurent Dortet

**Affiliations:** Bicêtre Hospital Bacteriology Hygiene, Le Kremlin-Bicetre, France (A.B. Jousset, J.-F. Mariet, C. Emeraud, T. Naas, R.A. Bonnin, L. Dortet);; Institut National de la Santé et de la Recherche Médicale, Paris, France (A.B. Jousset, L. Bouabdallah, S. Oueslati, C. Emeraud, D. Girlich, T. Naas, R.A. Bonnin, L. Dortet);; Centre National de Référence de la Résistance aux Antibiotiques, Le Kremlin-Bicetre (A.B. Jousset, J.-F. Mariet, C. Emeraud, T. Naas, R.A. Bonnin, L. Dortet);; Centre National de Référence de la Résistance aux Antibiotiques, Clermont-Ferrand, France (A. Birer);; Institut Pasteur, Paris (I. Rosinski-Chupin, P. Glaser);; Universite Paris-Sud, Paris (T. Naas)

**Keywords:** Escherichia coli, bacteria, antimicrobial resistance, enteric infections, food safety, carbapenemase, cefiderocol, Enterobacterales, epidemiology, France

## Abstract

Cefiderocol resistance is increasingly reported in New Delhi metallo-β-lactamase–producing Enterobacterales. Genomic and phenotypic analysis of *Escherichia coli* sequence type 361, a primary clone causing carbapenemase spread in France, revealed mutations leading to cefiderocol resistance. Continued genomic surveillance of carbapenem-resistant Enterobacterales could clarify prevalence of cefiderocol-resistant *E. coli* in Europe.

Few last-line antimicrobial agents effectively treat infections caused by New Delhi metallo-β-lactamase (NDM)–producing Enterobacterales (*1*). Cefiderocol is a novel synthetic conjugate siderophore cephalosporin that is more stable against β-lactamase hydrolysis than classical cephalosporins (*2*). However, several acquired cefiderocol-resistance mechanisms have been described in Enterobacterales, including increased *bla*_NDM_ copy numbers (*3*), specific *bla*_KPC_ variants (*4*), structural change in AmpC (*5*), and mutations or inactivation of siderophore receptors (*6*). Specific polymorphisms in penicillin-binding protein 3 (PBP3), the target of cefiderocol, also have been reported in *Acinetobacter* and *Escherichia coli* (*7**–**9*). However, prevalence of those polymorphisms and effects of cumulative resistance mechanisms have not been fully evaluated.

Since 2012, the French National Reference Center (F-NRC) for Antimicrobial Resistance has conducted active nationwide surveillance of carbapenemase-producing Enterobacterales (CPE). In 2022, the percentage of *E. coli* sequence type (ST) 361 isolates sent to F-NRC doubled to 1.2% from 0.6% of CPE in 2021. We characterized emerging *E. coli* ST361 in France and investigated cefiderocol resistance among CPE.

## The Study

**Figure 1:**
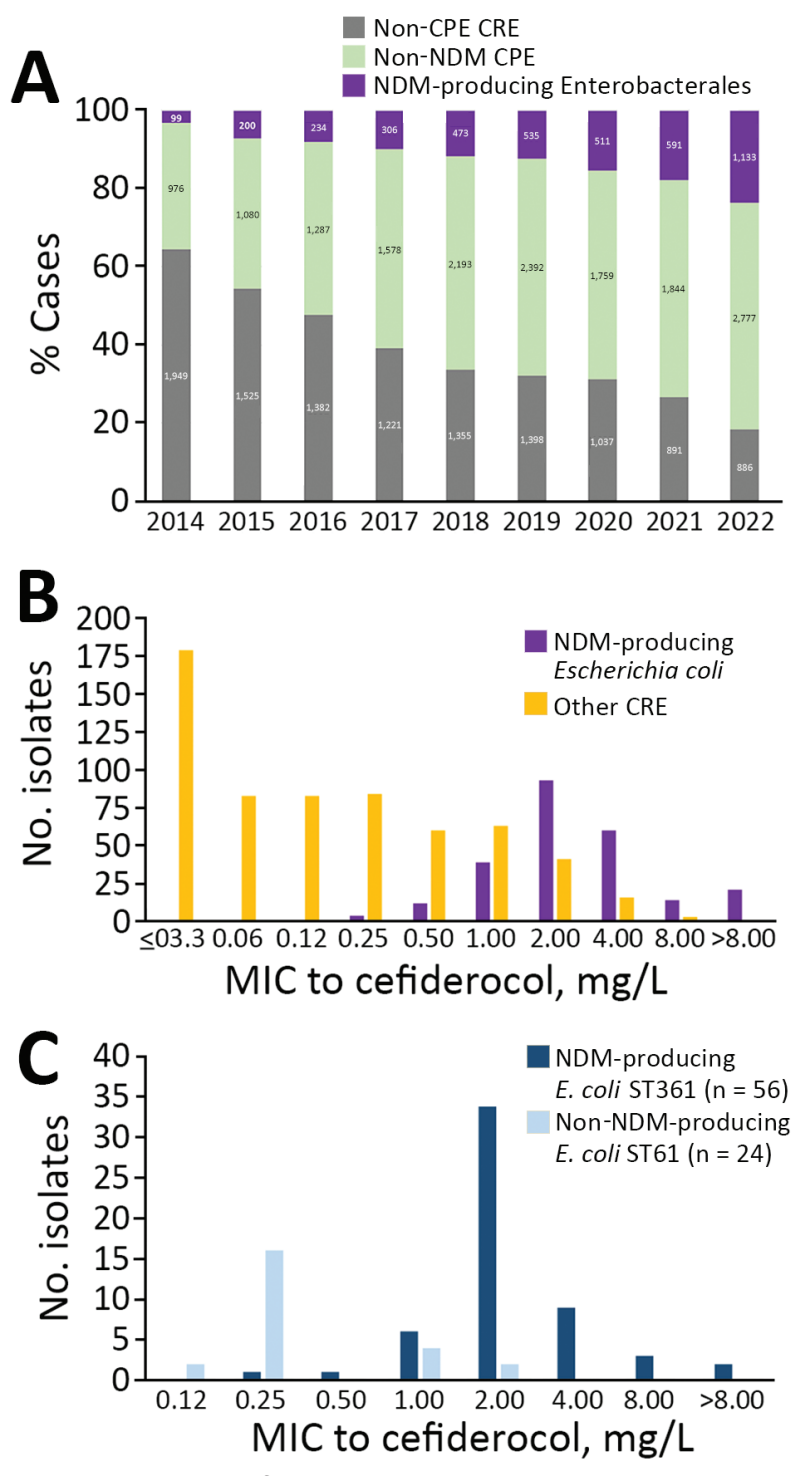
Evolution of NDM-producing and non–NDM-producing CPE observed in a population analysis of *Escherichia coli* ST361 and reduced cefiderocol susceptibility, France. A) Evolution of non-CPE CRE, non-NDM CRE, and non–NDM-producing Enterobacterales sent to the French National Reference Center for Antimicrobial Resistance during 2014–2022. B) Distribution of cefiderocol MICs in all (n = 856) CRE isolates collected during the study, July 1, 2021–June 30, 2022. C) Distribution of cefiderocol MICs in all (n = 80) *E. coli* ST361 isolates from the French National Reference Center for Antimicrobial Resistance collection, 2015–2022. CPE, carbapenemase-producing Enterobacterales; CRE, carbapenem-resistant Enterobacterales; NDM, New Delhi metallo-β-lactamase; non-CPE, non–carbapenemase producing; non-NDM CPE, non–NDM carbapenemase-producing Enterobacterales; ST, sequence type.

Since 2014, prevalence of NDM-producing Enterobacterales has been increasing in France (Figure 1, panel A). Among NDM producers, we observed a polyclonal dissemination of *E. coli* isolates, but 50% of isolates were from 4 main clones (ST410, ST167, ST361, and ST405), as reported in other countries in Europe (Appendix 1 Figure 1) (*10*). *E. coli* ST410, ST167, and ST405 have been characterized at the genomic level (*3*,*8*,*11*), but ST361 characteristics remain unclear.

**Figure 2:**
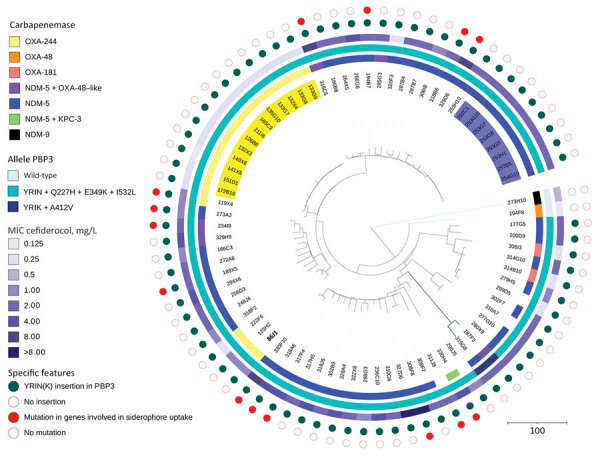
Phylogenetic analysis of 80 *Escherichia coli* ST361 isolates collected during 2015–2022 and used in a population analysis of *E. coli* ST361 and reduced cefiderocol susceptibility, France. Isolates were sent to the French National Reference Center for carbapenem-resistant Enterobacterales testing as part of routine surveillance. The phylogenetic tree was built by using SNIppy version 4.6.0 (https://github.com/tseemann/snippy) on whole-genome sequences. Data were visualized using iTOL 6.5.2 (https://itol.embl.de). The most ancient isolate, isolate no. 86J1 collected in 2015 (bold text, lower left of tree), was used as reference genome. A total of 4,957,882 nt positions were analyzed in the comparison. Colors indicate various outbreaks involving 13 OXA-244 producers (yellow) and 7 NDM-5 producers (blue). Two specific features are represented with filled circles: a YRIN(K) insertion in PBP3 (green) and chromosomal mutations within genes involved in siderophore-iron uptake (red). Genes investigated were *cirA, fiu, fepA, fepB, fecA, fhuA, tonB, pcnB, exbB, exbD, baeS/baeR,* and *ompR/envZ*. Scale bar indicates nucleotide substitutions per site. KPC, *Klebsiella pneumoniae* carbapenemase; NDM, New Delhi metallo-β-lactamase; OXA, oxacillinase; PBP3, penicillin-binding protein 3.

During July 1, 2021–June 30, 2022, we investigated all (n = 856) nonduplicate carbapenem-nonsusceptible *E. coli* isolates sent to F-NRC. We used Sensititer broth microdilution (ThermoFisher, https://www.thermofisher.com), as previously described (*12*), to measure MICs of aztreonam, ceftazidime-avibactam, imipenem, meropenem, and cefiderocol (Figure 2). Of note, the Mueller–Hinton broths used were from batches not affected by the manufacturer’s withdrawal relayed by European Committee on Antimicrobial Susceptibility Testing (https://www.eucast.org/ast-of-bacteria/warnings). Among tested isolates, 774 were CPE, including 243 NDM producers. The MIC_50_ (MIC to inhibit growth of 50% of isolates) of cefiderocol was higher (2 mg/L) for NDM producers among isolates tested compared with other carbapenem-resistant *E. coli* (0.12 mg/L) (Figure 1, panel B), as previously reported (*12*).

To genomically characterize *E. coli* ST361, we added all (n = 51) ST361 isolates sent to F-NRC during 2015–2021 to the 29 isolates collected during the study period. We conducted short-read sequencing on those 80 isolates by using the NextSeq500 system (Illumina, https://www.illumina.com). We assembled sequences by using Shovill 1.1.0 (https://github.com/tseemann/shovill) and SPAdes 3.14.0 (https://github.com/ablab/spades) under GenBank BioProject no. PRJNA925451 (Appendix 2 Table 1). We used Resfinder 4.1 (*13*) to analyze resistome content and PlasmidFinder 2.1 (*14*) to analyze replicon content (Appendix 2 Table 2).

Among 80 *E. coli* ST361 isolates, 50 produced NDM carbapenemase, 49 of which were NDM-5; another 20 produced oxacillinase 48–like carbapenemase; 6 coproduced NDM-5 with another carbapenemase; and 4 did not produce carbapenemase (Figure 2). Analysis of cefiderocol MIC distribution for ST361 showed that isolates with MICs >2 produced NDM, but that analysis also suggested that mechanisms besides NDM are involved in cefiderocol resistance (Figure 1, panel C). Thus, we analyzed the *bla*_NDM_ gene copy number on CLC Genomics Workbench 21.0 (QIAGEN, https://www.qiagen.com), where we mapped the raw data (fastq reads) on the genome (fasta) of each corresponding *E. coli* sequence. Then we normalized the average coverage of *bla*_NDM_ mapping reads to the average coverage of 10 different chromosomal genes used as references. However, correlation analysis did not reveal an association between *bla*_NDM_ gene number and the cefiderocol MIC (data not shown). Then we used *E. coli* K-12 MG1655 (GenBank accession no. NC_000913) as a reference to investigate *cirA*, *fiu*, *fepA*, *fepB*, *fecA*, *fhuA*, *tonB*, *pcnB*, *exbB*, *exbD*, *baeS/baeR*, and *ompR/envZ* gene mutations involved in siderophore-iron uptake. To eliminate polymorphisms linked to the ST itself, we only considered amino acid substitutions not shared by all ST361 isolates. A total of 14 (18%) isolates displayed a mutation in 1 of those genes (Appendix 2 Table 1). Overall, analysis of variance multiple parameter correlation analysis in RStudio 2022.07.1 (The R Foundation for Statistical Computing, https://www.r-project.org) revealed that *bla*_NDM_ (p = 0.0035) or chromosomal mutations (p = 0.0033) within a siderophore receptor were associated with higher cefiderocol MICs.

We also analyzed the preferential cefiderocol target, PBP3. That analysis revealed that compared with the reference, 76 isolates shared a common allele that had a 4 amino acid insertion (YRIN motif) at position 333 and 3 substitutions (Q227H, E349K, and I532L). Two isolates had a different allele with a YRIK insertion and an A412V substitution, and 2 isolates had no insertions or mutations. Of note, the 3 different alleles were associated with 3 different nodes on the phylogenetic tree, indicating an evolution process that probably involved chromosomal recombination (Figure 2), as described for ST410 (*11*). The YRIN(K) motif insertion has been described to be involved in cephalosporin and aztreonam resistance (*8*,*9*,*11*). To study the effect of the YRIN(K) motif insertion on cefiderocol resistance, we performed susceptibility testing on the reference strain and its isogenic PBP3 encoding gene mutant with YRIN insertion (*11*). We transformed both strains by plasmid topoisomerase-based cloning *bla*_NDM-1_ to increase the basal range of cefiderocol MIC concentrations in the microbroth dilution technique. The YRIN insertion resulted in a 4-fold increase in cefiderocol MIC, from <0.03 mg/L to 0.125 mg/L in the YRIN *ftsI* chromosomal mutant.

We also analyzed all (n = 321) available ST361 genomes and metadata in EnteroBase (University of Warwick, https://warwick.ac.uk/fac/sci/med/research/biomedical/mi/enterobase) on October 1, 2022 (Appendix 1 Figures 3, 4; Appendix 2 Table 3). The 401-isolate phylogenetic tree showed that isolates from F-NRC were distributed within several main branches (Appendix 1 Figure 3), confirming our collection’s diversity. Of note, the YRIN(K) insertion occurred in only 36% of the EnteroBase genomes but occurred in 97% of NDM producers; however, only 7% of non–NDM producers had the modified alleles. The phylogenetic tree enabled visualization of this strong association between occurrence of NDM and PBP3 alleles possessing the YRIN(K) insertion. Furthermore, specifying isolate locations revealed international ST361 circulation.

We also examined genomes sequenced at F-NRC during 2015–2022 that are from 3 other predominant STs disseminating NDM-5 in France. Among those genomes, we noted a high prevalence of YRIN(K) insertion in PBP3, namely in 98% of ST410 (n = 273), 92% of ST167 (n = 184), and 86% of ST405 (n = 122), regardless of β-lactamase content (Appendix 1 Figure 5, panel A). YRIN(K) insertion prevalence was only 4% in *E. coli* ST131 (n = 166), another high-risk clone associated with multiple β-lactamases (*15*). Distribution analysis of cefiderocol MICs in ST410, ST167, and ST405, excluding NDM-producing isolates, revealed a MIC_50_ of 1 mg/L, confirming the role of the genetic background in reduced cefiderocol susceptibility (Appendix 1 Figure 5, panel B).

## Conclusions

Our results highlight the emergence of NDM-producing *E. coli* ST361 associated with reduced cefiderocol susceptibility in France. Emergence resulted from a combination of factors: modified PBP3, a strong association with NDM-5 carbapenemase, and frequent chromosomal mutations in genes involved in siderophore-iron uptake. No feature alone is sufficient to confer cefiderocol resistance, according to published clinical breakpoints (https://www.eucast.org/clinical_breakpoints), but the combined mechanisms appear to confer resistance. 

In conclusion, our study revealed that *E. coli* ST361 is becoming a key player in NDM-5 carbapenemase dissemination, and its genetic background confers reduced cefiderocol susceptibility. *E. coli* ST361 has only been sporadically reported, but its prevalence might be underestimated. To further assess prevalence and spread of cefiderocol-resistant *E. coli* in Europe, each country should continue nationwide genomic surveillance of carbapenemase-resistant bacteria.

Appendix 1 Additional information on population analysis of *Escherichia coli* ST361 and reduced cefiderocol susceptibility, France.

Appendix 2 Additional metadata and genomic data used in a population analysis of *Escherichia coli* ST361 and reduced cefiderocol susceptibility, France.
