# Supplementary material for: Population Analysis of Escherichia coli Sequence Type 361 and Reduced Cefiderocol Susceptibility, France
Source: Emerg Infect Dis. 2023 Sep;29(9):1877–81. doi: 10.3201/eid2909.230390 (PMC10461684; doi:10.3201/eid2909.230390)
Supplement: Appendix 1 — Additional information on population analysis of Escherichia coli ST361 and reduced cefiderocol susceptibility, France. [file 23-0390-Techapp-s1.pdf]

Article DOI: https://doi.org/10.3201/eid2909.230390

EID cannot ensure accessibility for supplementary materials supplied by authors. Readers who have difficulty accessing supplementary content should contact the authors for assistance.

Population Analysis of *Escherichia coli* ST361 and Reduced Cefiderocol Susceptibility, France

Appendix 1

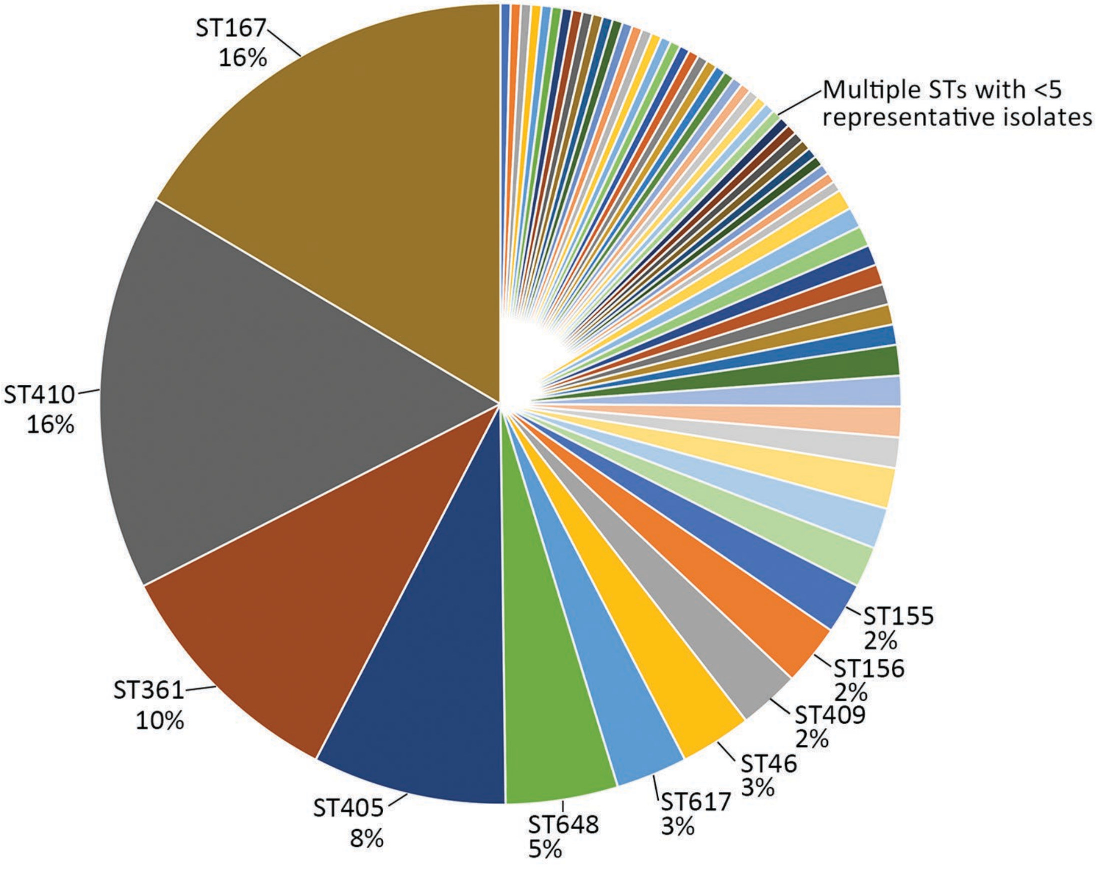

Appendix 1 Figure 1. Sequence types of 243 NDM-producing *Escherichia coli* isolates collected in a population analysis of *E. coli* ST361 and reduced cefiderocol susceptibility, France. Isolates were collected by the French National Reference Center during July 1, 2021–June 30, 2022. NDM, New Delhi metallo-β-lactamase; ST, sequence type.

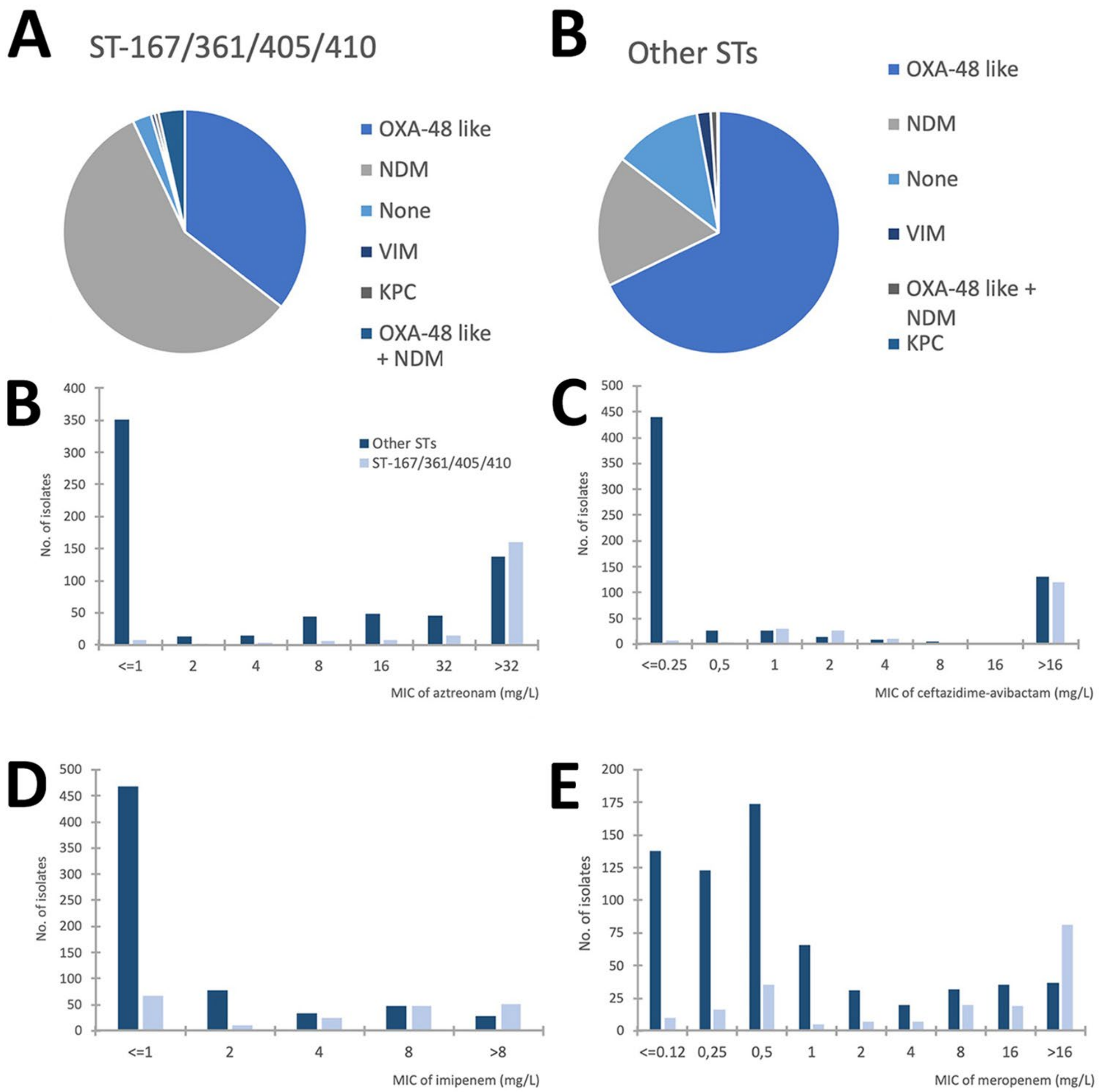

Appendix 1 Figure 2. Distribution of antimicrobial resistance genes and MICs to antimicrobial drugs among a collection of 856 carbapenem-resistant *Escherichia coli* isolates sent to the French National Reference Center during July 1, 2021–June 30, 2022. A,B) Pie charts show distribution of resistance genes in ST167, ST361, ST405, and ST410 (A) and in other STs (B). C–F) MICs for individual antimicrobial drugs; C) aztreonam; D) ceftazidimine-avibactam; E) Imipenem; F) Meropenem. ST, sequence type.

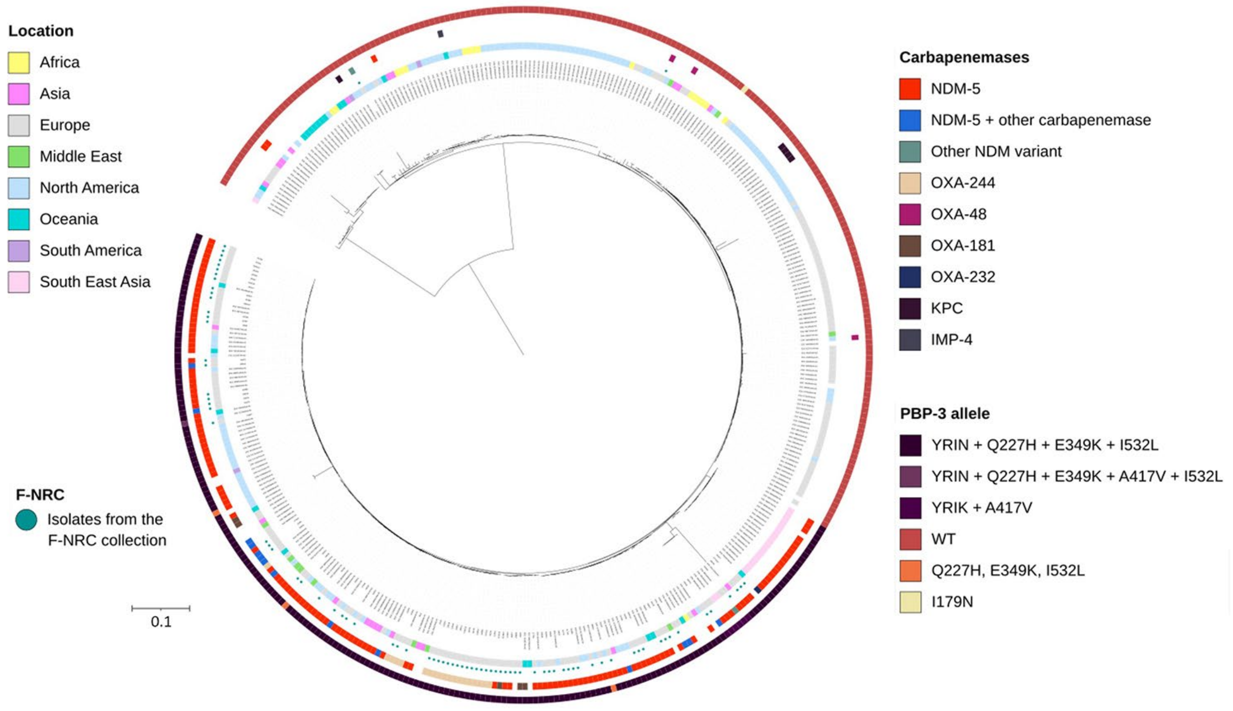

Appendix 1 Figure 3. Phylogenetic analysis *Escherichia coli* ST361 in a study of cefiderocol-resistant *E. coli*, France. Data were visualized using iTOL 6.5.2 (https://itol.embl.de). The tree includes 80 *E. coli* ST361 isolates sent to F-RNC and 321 *E. coli* ST361 genomes recovered from EnteroBase (University of Warwick, https://warwick.ac.uk/fac/sci/med/research/biomedical/mi/enterobase). Genome ESC_LB4496AA_AS was used as reference. The tree is mid-point rooted. The inner circle indicates the location of isolate collection according to the metadata declared in EnteroBase. Scale bar indicates nucleotide substitutions per site. F-RNC, French National Reference Center; KPC, *Klebsiella pneumoniae* carbapenemase; NDM, New Delhi metallo-β-lactamase; OXA, oxacillinase; PBP-3, penicillin binding protein 3.

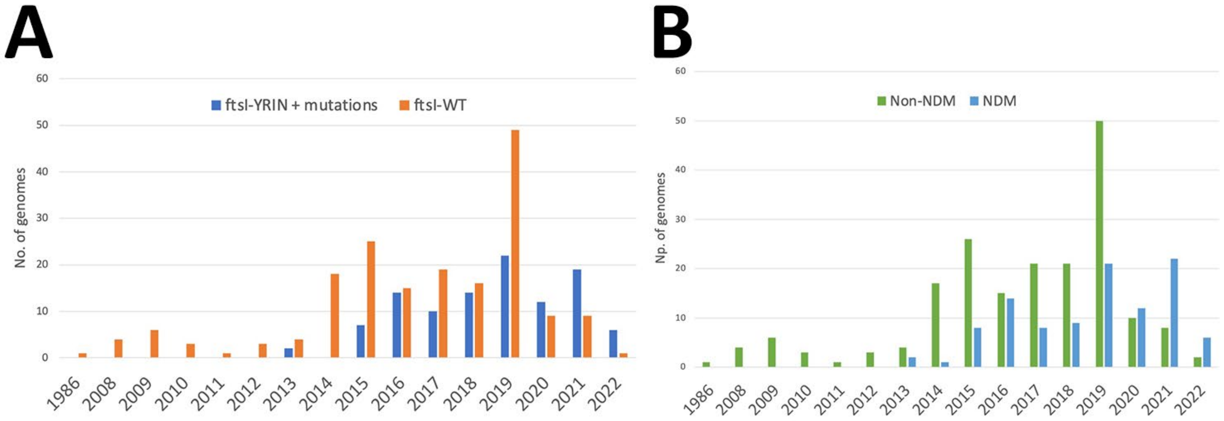

Appendix 1 Figure 4. Distribution of 2 main *ftsI* alleles and NDM carbapenemase in 361 *Escherichia coli* ST361 genomes according to isolation date use in a study of cefiderocol-resistant *E. coli*, France. Data are from the EnteroBase database (University of Warwick, https://warwick.ac.uk/fac/sci/med/research/biomedical/mi/enterobase). A) *ftsI* alleles; B) NDM carbapenemase. NDM, New Delhi metallo-β-lactamase.

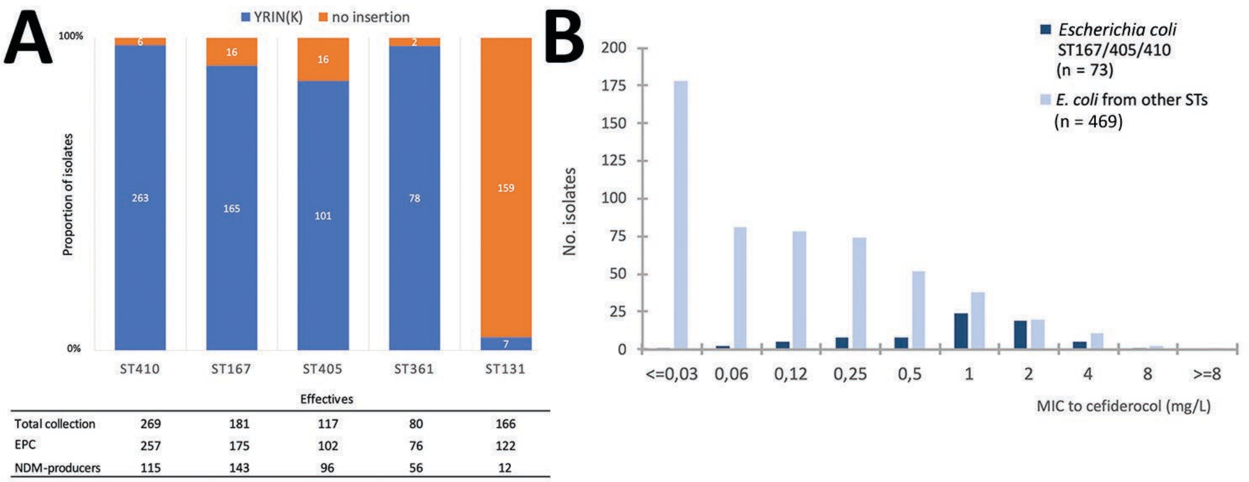

Appendix 1 Figure 5. Analysis of prevalence of YRIN(K) motif insertion in PBP3 and MICs of *Escherichia coli* isolates submitted to the F-NRC. A) Analysis of prevalence of YRIN(K) motif insertion in PBP3 in all *E. coli* ST410, ST167, ST361, and ST131 genomes sequenced by the F-NRC during 2015–2022. Data include NDM-producers and total EPC. B) Distribution of cefiderocol MICs of carbapenem-resistant *E. coli* isolates collected during July 1, 2021–June 30, 2022; 243 NDM-producing isolates were excluded from the total dataset with available ST (n = 789). EPC, *E. coli* carbapenemase-producers F-NRC, French National Reference Center; NDM, New Delhi metallo-β-lactamase; PBP3, penicillin binding protein 3.
